# Study protocol: cross-national comparative case study of recovery-focused mental health care planning and coordination (COCAPP)

**DOI:** 10.1186/s12888-015-0538-2

**Published:** 2015-07-03

**Authors:** Alan Simpson, Ben Hannigan, Michael Coffey, Aled Jones, Sally Barlow, Rachel Cohen, Jitka Všetečková, Alison Faulkner, Mark Haddad

**Affiliations:** 1 Centre for Mental Health Research, School of Health Sciences, City University London, London, UK; 2 School of Healthcare Sciences, Cardiff University, Cardiff, UK; 3 Department of Public Health and Policy Studies, Swansea, UK; 4 Faculty of Health and Social Care, The Open University, Milton Keynes, UK; 5 Independent Service User Researcher Consultant, London, UK

**Keywords:** Care planning, Care and treatment planning, Recovery, Care coordination, Personalisation

## Abstract

**Background:**

The collaborative care planning study (COCAPP) is a cross-national comparative study of care planning and coordination in community mental healthcare settings. The context and delivery of mental health care is diverging between the countries of England and Wales whilst retaining points of common interest, hence providing a rich geographical comparison for research. Across England the key vehicle for the provision of recovery-focused, personalised, collaborative mental health care is the care programme approach (CPA). The CPA is a form of case management introduced in England in 1991, then revised in 2008. In Wales the CPA was introduced in 2003 but has now been superseded by The Mental Health (Care Co-ordination and Care and Treatment Planning) (CTP) Regulations (Mental Health Measure), a new statutory framework. In both countries, the CPA/CTP requires providers to: comprehensively assess health/social care needs and risks; develop a written care plan (which may incorporate risk assessments, crisis and contingency plans, advanced directives, relapse prevention plans, etc.) in collaboration with the service user and carer(s); allocate a care coordinator; and regularly review care. The overarching aim of this study is to identify and describe the factors that ensure CPA/CTP care planning and coordination is personalised, recovery-focused and conducted collaboratively.

**Methods/design:**

COCAPP will employ a concurrent transformative mixed methods approach with embedded case studies. Phase 1 (Macro-level) will consider the national context through a meta-narrative mapping (MNM) review of national policies and the relevant research literature. Phase 2 (Meso-level and Micro-level) will include in-depth micro-level case studies of everyday ‘frontline’ practice and experience with detailed qualitative data from interviews and reviews of individual care plans. This will be nested within larger meso-level survey datasets, senior-level interviews and policy reviews in order to provide potential explanations and understanding.

**Discussion:**

COCAPP will help identify the key components that support and hinder the provision of personalised, recovery-focused care planning and provide an informed rationale for a future planned intervention and evaluation.

## Background

### Cross-national approaches to care planning

Following the Government of Wales Act (2006) and subsequent devolution of certain powers, the context and delivery of mental health care is diverging between the countries of England and Wales whilst retaining points of common interest, hence providing a rich geographical comparison for research. Across England the key vehicle for the provision of recovery-focused, personalised, collaborative mental health care is the care programme approach (CPA). The CPA is a form of case management introduced in England in 1991, then revised and refocused [1]. In Wales the CPA was introduced in 2003 [2] but has now been superseded by The Mental Health (Care Co-ordination and Care and Treatment Planning) (CTP) Regulations (Mental Health Measure), a new statutory framework [3]. Data for England shows that 403,615 people were on the CPA in 2011/12 [4]. Centrally-held CPA numbers supplied by the Corporate Analysis Team at the Welsh Government indicate 22,776 people in receipt of services as of December 2011, just six months prior to the introduction of CTP under the Mental Health Measure.

In both countries, the CPA/CTP requires providers to: comprehensively assess health/social care needs and risks; develop a written care plan (which may incorporate risk assessments, crisis and contingency plans, advanced directives, relapse prevention plans, etc.) in collaboration with the service user and carer(s); allocate a care coordinator; and regularly review care. Both CPA and CTP processes are now also expected to reflect a philosophy of *recovery* and to promote *personalised* care [1, 5], although interpretations of personalisation may vary between countries [6].

### Recovery and personalisation

The concept of recovery in mental health was initially developed by service users and has led to disparate conceptualisations [7] but broadly refers to “a way of living a satisfying, hopeful, and contributing life even with limitations caused by illness,” while developing new purpose or meaning [8] ^(p527)^. The importance of addressing service users’ *personal recovery*, alongside more conventional ideas of *clinical recovery* [9] is now supported in guidance for all key professions [10–13]. To this has been added the more recent idea of *personalisation*. Underpinned by recovery concepts, this aims to see people and their families taking much more control over their own support and treatment options, alongside new levels of partnership and collaboration between service users and professionals [14]. *Recovery* and *personalisation* in combination mean practitioners tailoring support and services to fit the specific needs of the individual and enabling social integration through greater involvement of local communities.

### Implementation of care planning procedures

Cochrane Collaboration systematic reviews of case management including the CPA [15] did not consider recovery-oriented outcomes and few studies are explicitly conducted into the practices of CPA care planning and coordination. Early investigations in England prior to the refocus on recovery drew attention to the bureaucracy associated with care coordination which, combined with high caseloads, deflected practitioners from therapeutic interventions linked to positive outcomes [16, 17].

National audits in England reported considerable local variation in implementation of the CPA, and despite improvements in performance, significant numbers of service users not receiving care in line with guidelines [18]. A review conducted in Wales reflected concerns in: risk assessment, care planning, unmet need and service planning, training, information requirements and systems, transfer of care arrangements, and leadership [19]. The authors concluded there was a high risk that services were not effectively meeting users’ and carers’ needs and that significant improvement was required.

### Service user views on care planning

Service users appear to be often mystified by the care planning and review processes. In a national quality survey of over 17,000 community mental health service users across 65 English National Health Service (NHS) Trusts, 42 % said that their care was coordinated under the CPA [20]. Over 90 % of all respondents described their care as well organised and 83 % of those on the CPA knew who their care coordinators were. Despite this, over half did not understand their care plans; only 16 % had written copies; 20 % said their care plans did not set out their goals; and 11 % said their views had not been taken into account during care planning. In Wales, 310 users of NHS/local authority mental health services responded to a similar survey [21]. Only 58 % knew who their care coordinator was; just half were given or offered copies of their care plans, with only 51 % ‘definitely’ understanding the content of care plans and 43 % ‘definitely’ involved in ‘co-producing’ the content.

The need for greater co-production has also been found in the area of risk management. Research for the Joseph Rowntree Foundation [22] on service users’ views on risk reported that perceptions of risk and rights were significantly different for mental health service users. Practitioners tended to perceive people as a source of risk first rather than being considered potentially at risk in vulnerable situations; they appeared to be overlooked by adult safeguarding practices; and their individual rights were compromised by mental health legislation.

This evidence, which points to the relative lack of genuine service user involvement in CPA/CTP processes, is significant in the context of what we know about therapeutic relationships and recovery. The therapeutic relationship is a reliable predictor of patient outcomes in mainstream psychiatric care [23, 24]. Strong, collaborative, working alliances between case managers and people with long-term mental health difficulties have been shown to reduce symptoms, improve levels of functioning and social skills, promote quality of life, enhance medication compliance and raise levels of satisfaction with care received [25]. Yamashita et al. [26] describe negotiating care within a trusting relationship as key in case management and this relationship may influence users’ perceptions of stigma [27].

The limited available evidence contrasts with the aspiration that CPA/CTP care planning and related processes should be collaborative, personalised and recovery-oriented. In addition, the current approach to assessing and managing risk under the CPA may not be satisfactory for either service providers or service users. This study aims to address this lack of evidence and hopes to inform the development of a complex intervention aimed at delivering, recovery-focused care planning and coordination and improved patient outcomes.

### Study aims

The study has six related aims:

1. Review the international peer-reviewed literature on personalised recovery-oriented care coordination, and compare and contrast the English and Welsh contexts for recovery-based mental health care.
2. Conduct a series of case studies to examine in detail how the needs of people with severe mental illness using community mental health services are assessed, planned and coordinated.
3. Investigate service users’, informal carers’, practitioners’ and managers’ views of these processes and how to improve them in line with a personalised, recovery-oriented focus.
4. Measure service user and staff perceptions of recovery oriented practices.
5. Measure service users’ views of empowerment and the quality of therapeutic relationships.
6. Identify methods, measures and processes for successfully evaluating a complex intervention aimed at delivering personalised, recovery-focused care planning and coordination and improved patient outcomes.

## Methods

### Study design

The Collaborative Care Planning Project (COCAPP) will use a concurrent transformative mixed methods approach with embedded case studies [28]. The study will use simultaneous procedures to collect quantitative and qualitative data at the same time and integrate the data to provide a comprehensive analysis of the research problem.

**Fig. 1:**
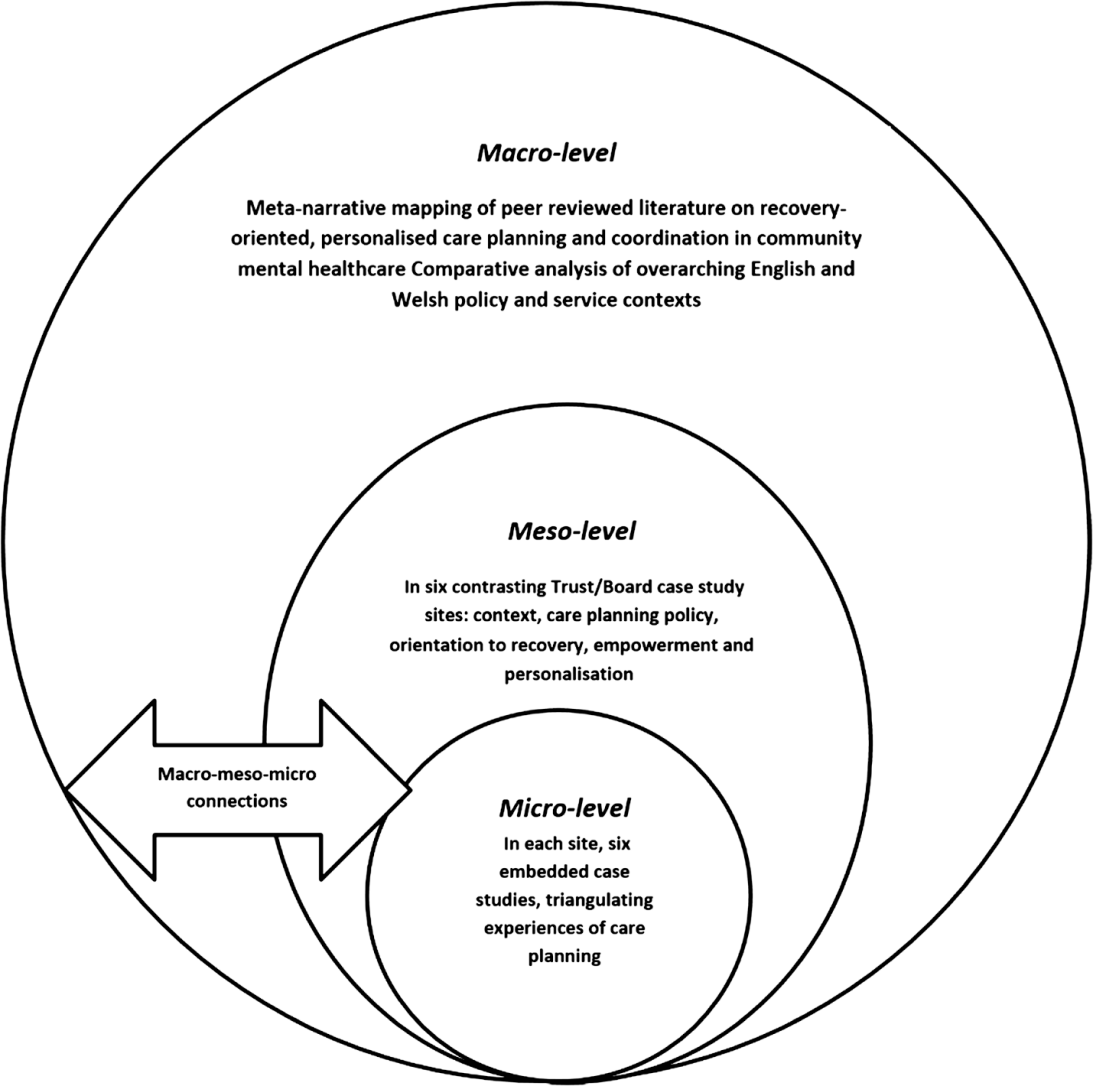
Diagram of study design with embedded case studies

In this study, in-depth micro-level case studies of everyday ‘frontline’ practice and experience with detailed qualitative data from interviews and reviews of individual care plans will be nested within larger meso-level survey datasets, senior-level interviews and policy reviews in order to provide potential explanations and understanding (see Fig. 1).

At the macro-level is the national context. Cross-national comparative research involves “comparisons of political and economic systems …and social structures” [29] ^(p93)^ where “one or more units in two or more societies, cultures or countries are compared in respect of the same concepts and concerning the systematic analysis of phenomena, usually with the intention of explaining them and generalising from them” [30] ^(p1–2)^. In this study, devolved government and the emergence of similar but distinct health policy, legislation and service development in England and Wales will provide a fascinating backdrop for the investigation of community mental health care.

Such an approach fits well with a case study method [31] that allows the exploration of a particular phenomenon within dynamic contexts where multiple influencing variables are difficult to isolate [32]. It allows consideration of historical and social contexts [33] and is especially useful in explaining real-life causal links that are maybe too complex for survey or experimental approaches [34]. In this study, we will conduct a detailed comparative analysis of ostensibly similar approaches to recovery-focused care planning and coordination within different historical, governmental, legislative, policy and provider contexts in England and Wales.

### Theoretical/conceptual framework

Transformative research seeks to include an explicit “intent to advocate for an improvement in human interests and society through addressing issues of power and social relationships” [35] ^(p441)^. In line with this, transformative procedures require the researcher to employ a transformative theoretical lens as an overarching perspective [36]. This lens provides a framework for topics of interest, methods for collecting data, and outcomes or changes anticipated by the study. In our study, our choice of methods, data collection and approach to analysis is guided by a theoretical framework emphasising the connections between different ‘macro/meso/micro’ levels of organisation [37] and concepts of recovery and personalisation that foreground the service user perspective and, arguably, may challenge more traditional service/professional perspectives. Furthermore, our research team and processes will involve mental health service users throughout.

**Fig. 2:**
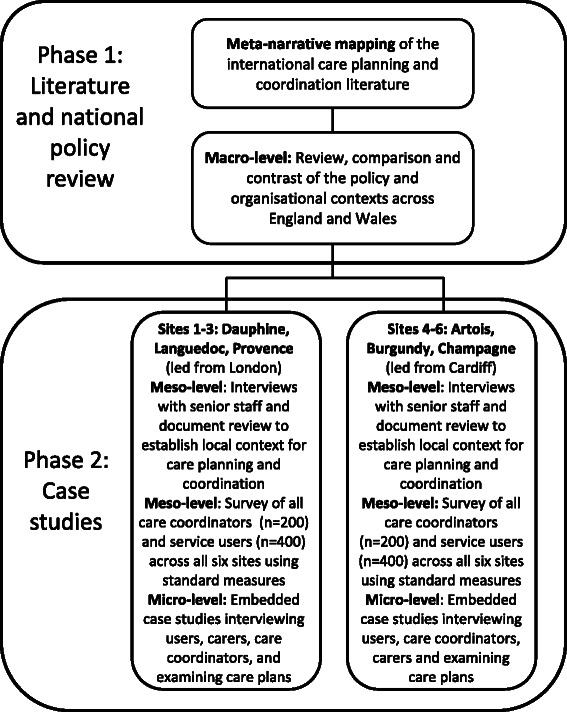
Diagram of study plan

**Fig. 3:**
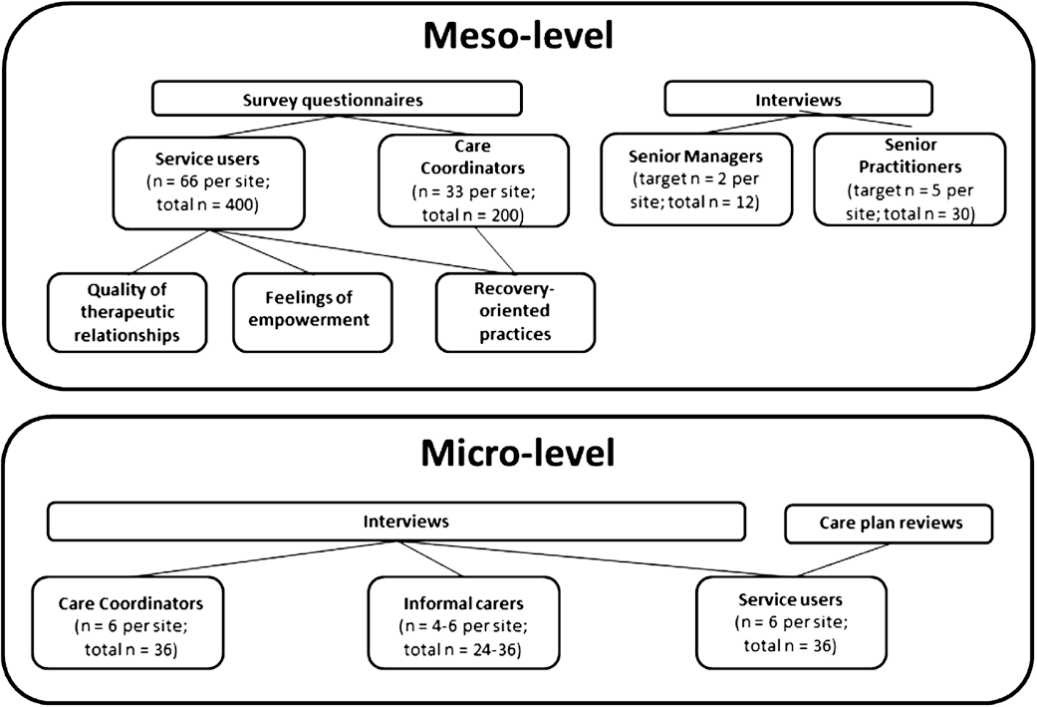
Sample size and data collection targets

The study will incorporate two phases; phase 1 focuses on methodology to collect macro-level data and phase 2 will focuses on the collecting meso and micro-level data (see Fig. 2 and Fig. 3). The content is outlined below:

1. **Phase 1**: we will conduct a) a review of international literature on care planning and coordination processes and their relationships to recovery and personalisation; and b) a comparative analysis of mental health policy and service frameworks in England and Wales.
2. **Phase 2:** we will conduct six in-depth case study investigations [31] across contrasting case study sites in England (n=4) and Wales (n=2).

### Phase 1: Literature and policy review and synthesis

#### Macro-level – English and Welsh contexts

##### Literature review on mental health care planning and coordination processes

We will use Greenhalgh et al’s [38] meta-narrative mapping technique (MNM), which focuses on providing a review of evidence that is most useful, rigorous and relevant for service providers and decision-makers and that integrates a wide range of evidence [39]. Our MNM review will provide a preliminary map of current mental health care planning and coordination by addressing four points: 1) how the topic is conceptualised in different research traditions; 2) what the key theories are; 3) what the preferred study designs and approaches are; and 4) what the main empirical findings are.

##### Search strategy

Initial literature searches will be undertaken using the following key words and terms: ‘mental health’, ‘care planning’, ‘care coordination’ (and ‘coordination’), ‘collaborative care’, ‘recovery’, ‘recovery focus(ed)’, personali*. These preliminary searches will reveal relevant papers and inform additional search terms/phrases. The search strategy will be discussed within the team and modifications made accordingly. We anticipate that the number of search terms in the strategy will expand to reflect the terminology used within clinical practice and relevant research studies. The Lived Experience Advisory Group (LEAG) and the Project Advisory Group (PAG) will also be consulted and further search terms may be identified. Proximity indicators (such as ADJ or N- as appropriate of each database), truncation ($) and wildcard (*) symbols as well as Boolean commands (AND and OR) will be used where appropriate. Key search terms will also be searched by their subject (MeSH headings) and by keyword searches. We will use the following databases: ASSIA, CINAHL, AMED, EMBASE, the Cochrane library, Medline, PsycINFO, ERIC, BHI, Scopus, Social Care Online and Web of Science.

The inclusion criteria for the search are:

- Studies from 1990 (at introduction of the CPA) to 2014.
- Articles in the English language only.

The search will be replicated in two of the databases ASSIA and CINAHL in order to verify the strategy. This will be completed by an independent information specialist (health and social care librarian) working for the Information Sciences service at Cardiff University.

Duplicate cases will be removed and the remaining references will be retrieved and entered into an EndNote library. These references will be screened by two members of the research study team to identify key papers for the meta-narrative synthesis. The focus will be on papers about care planning and coordination in mental health in the community. The papers will be labelled as Y-Yes, N-No and M-Maybe to indicate their relevance to the review. Papers will be discussed and agreements among the team on the papers excluded. A further snowball search on the web and using Google Scholar will ensue to determine if there are any further papers missed by the database searches. These papers will also be screened to determine relevance to the review using the same methodology labelled above. The papers labelled ‘Maybe’ will be interrogated further to determine if back-chaining will reveal any further papers.

The quality of the studies will be considered; however we will take a conservative approach in rejecting papers where research is considered of low quality but provide a broad representation of approaches and views. However, some studies will be excluded on the grounds of insufficient detail about the research process undertaken within each study [40, 41].

Selected papers will then be reviewed to identify, synthesise and chart how particular research traditions have unfolded over time and shaped the kind of questions being asked and the methods used to answer them.

##### Comparative analysis of policy and service frameworks

Through searching English and Welsh Government websites we will identify all key, current, national-level policy and guidance documents directly relating to mental health care planning and coordination across the two countries, along with those which relate directly to the promotion of recovery and the delivery of personalised care. Drawing on these we will produce a narrative synthesis identifying major themes and areas of policy convergence and divergence and use these materials to lay out the large-scale (or ‘macro-level’) national policy contexts to inform our case study research interviews.

### Phase 2: Case Studies and Large Scale Survey

#### Meso-level - Trust/Health Board level

##### Objectives

This meso-level phase will be conducted in parallel with the micro-level study, with four main objectives:

1. Measure service user and staff perceptions of recovery orientated practices (Survey).
2. Measure service users’ views of empowerment and the quality of therapeutic relationships (Survey).
3. Investigate the subjective view of senior managers and senior practitioners regarding CPA care planning and coordination, recovery and personalisation (Semi-structured interviews).
4. Identify and review the policy and contextual factors likely to impact on providing personalised, recovery-focused care planning and coordination (Semi-structured interviews and local policy document review).

## Design

The study will use a case study design including a survey of a large sample of service users and care coordinators and semi-structured interviews with senior managers and practitioners at four NHS Trusts in England and two local Health Boards in Wales that are commissioned to deliver community mental health services.

### Sampling of sites

NHS Trusts and Health Boards will be identified which reflect variety in geography and population and include a mix of rural, urban and inner city settings in which routine community care is provided to people with complex and enduring mental health problems from across the spectrum of need. The selection of six sites is a pragmatic decision, balancing variety of settings and populations with logistical and data management pressures in the time available.

In each Trust/Board site suitable local Community Mental Health Teams (CMHTs) will be identified with the assistance of local NHS collaborators. We will select a single team providing routine community mental health care to conduct the semi-structured interviews. We will subsequently use this information to build a recovery profile of this team. The criteria to determine the team’s eligibility to participate are as follows:

Inclusion criteria: providing community mental health care to adults, team manager in post, reasonable stable staffing, not due for merger or closure.

Agreement to participate in the study will be obtained from senior Trust/Health Board managers prior to seeking research governance approval alongside NHS ethics approval. Before data collection commences researchers will present the study to the clinical team, explaining the methodology and responding to any queries.

### Materials

Three sets of study materials will be developed by the study team in consultation with our Project Advisory Group and Lived Experience Advisory Group and drawing on the relevant literature.

1. An interview schedule for senior managers and practitioners will be developed. All interviews will aim to explore participants’ views and experiences of care planning and coordination, safety and risk, recovery and personalisation and the context within which these operated. Interview schedules will include lead questions with numerous prompts suggested for the interviewer. Schedules will be piloted with our service user researchers and amended if required.
2. Two questionnaire booklets will be developed, one for a postal survey to service users and one for care coordinators. Both booklets will include demographic information. Survey questionnaires will focus on recovery oriented practices (both groups), and quality of therapeutic relationships and feelings of empowerment (service users only).
3. A Framework matrix will be developed. We will use the interview schedules to build an analytical framework consisting of several matrices focusing on categories linked to our research questions. Matrices will focus on organisational background and developments, care planning, recovery, personalisation, and recommendations for improvement. Each matrix section will have an ‘other’ column for the inclusion of data-led emergent categories.

## Semi-structured interviews – senior managers and senior practitioners

### Participants and sampling

Two senior managers will be invited for interview based on their knowledge of the organisational knowledge of the team. This will be informed by the Clinical Studies Officers (CSO). A purposive sampling strategy will be used to identify key personnel in a senior practitioner role. This will include consultant psychiatrists, senior mental health nurses, psychologists, social workers and occupational therapists. Potential participants will be invited to participate in interviews and asked to obtain local policies and information (Local meso-level CPA policy and procedure documents, Care Quality Commission (CQC), national and local audits and reviews).

### Inclusion criteria

#### Senior managers

1. Participants will be from health and social care organisations with responsibility for CPA or CTP.

#### Senior practitioners

1. Participants will be senior clinical staff managing care coordinators and/or providing care under the CPA or CTP.

### Procedure

Senior manager and senior practitioner interviews will be conducted by academic researchers.

1. One-to-one, semi- structured interviews, using an interview schedule and lasting approximately one hour, will be conducted at a time convenient to the interviewee. Interviews will be audio-recorded with permission.

### Analysis

1. Audio-recorded interviews will be professionally transcribed verbatim and transcripts will checked against original recordings for accuracy. Any identifying information will be redacted, before being imported into QSR International’s NVivo 10 qualitative data analysis software for analysis using Framework method [42, 43].
2. Several transcripts will be read by all members of the research team to familiarise themselves with the data.
3. A series of framework matrices will be created in NVivo. Transcripts will be imported into NVivo and linked to the framework matrices.
4. Ten transcripts will be summarised and charted by two researchers using the matrices. The initial summarising will be checked and discussed by other members of the research team. Amendments to the matrices will be made before summarising and charting any further transcripts ensued.
5. Following an agreed format for notation and linking transcripts to the text the rest of the transcripts will be charted.
6. Researchers will read and check ten per cent of each other’s summarising against transcripts to ensure accuracy and consistency of approach.
7. Once all charting is completed, second-level summarising will be undertaken to further *précis* data and identify commonalities and differences within Trust/Health Board sites and groups, e.g. senior managers.

## 2.Questionnaires

### Participant and sampling

Service users from CMHT caseloads will be randomly selected for invitation to participate via the service provider team. Guidance will be sought from the MHRN/ NISCHR CRC, Clinical Studies Officers and checks with the clinical team to prevent inappropriate mailings (e.g. to recently deceased patients).

### Inclusion criteria

#### Service users

1. Over the age of 18 years of age
2. Under the care of CPA or CTP.
3. Had a minimum of six months contact with the service.

#### Care coordinators

1. Care coordinators providing care under the CPA or CTP.

Instrumentation:

1. ***The Recovery Self-Assessment Scale (RSA)*** [44] is designed to measure the extent to which recovery-oriented practices are evident in services. It is a 36-item self-administered questionnaire which will be completed in this study by service users and care coordinators. The scale addresses the domains of life goals, involvement, treatment options, choice and individually tailored services. The RSA has been tested for use with people with enduring and complex mental health problems and across a range of ethnic backgrounds.
2. ***The Scale To Assess the Therapeutic Relationship (STAR-P)*** [45] is a specifically developed, brief (12-item) scale that assesses therapeutic relationships in community psychiatry. It has good psychometric properties and is suitable for use in research and routine care. The subscales measure positive collaborations, positive clinician input and non-supportive clinician input in the patient version. This will be completed by service users.
3. ***The Empowerment Scale (ES)*** [46] is a 28-item questionnaire with five distinct subscales: self-esteem, power, community activism, optimism and righteous anger. Empowerment is strongly associated with recovery and this is the most widely used scale, with good psychometric properties. This will be completed by service users

### Procedure

#### Care coordinator questionnaires

1. Researchers will distribute information sheets and questionnaires to CMHT care coordinators and collate completed questionnaires.
2. Where the identified CMHT has insufficient numbers of care coordinators, a second (or third) team will be approached within the host site with the questionnaire survey.

#### Service user questionnaires

1. Questionnaire packs and invitations to participate in the survey will be distributed by post to service users. The pack will include a covering letter; an invitation to participate, the patient information sheet, the pack of three questionnaires and a demographic information sheet. The envelope will also include a brief description of the study in numerous languages with details of who to contact for more information in other languages. A freepost return envelope will be included. In line with evidence-based recommendations to maximise returns of postal surveys [47], questionnaires will be printed single-sided and envelopes will be stamped with ‘Private and Confidential’ and the University logo.
2. Reminder letters will be posted out to all recipients two to three weeks after the initial mail-out.

### Sample size and power calculation

The key variables of interest for this study are the responses of service users and healthcare staff in relation to the extent to which recovery-oriented practices are evident in the services surveyed. An established measure, the Recovery Self-Assessment Scale (RSA) [44] will be used for this purpose, and prior investigations among US mental health services provide mean and standard deviation values on which to base estimates using the standard formula for scaled and categorical items [48]. Findings from the prior study provided a range of mean values for the RSA summary score from mean (s.d.) 3.87 (0.62) (providers) to 4.06 (0.69) (people in recovery). Applying a 0.69 standard deviation value and an error margin or precision level of 3 % provided a total sample size of 127. Anticipating a potential non-response rate of 40 per cent requires inflation of the sample size to 250 to allow for this. In our study, we plan to seek RSA responses from service users (n=400) and care coordinators (n=200); these calculations indicate that generalisability to the target population and appropriate precision in findings is likely, even in the event of a poor survey response rate.

### Micro-level (Embedded Case Studies)

#### Design

The study will use a case study design involving semi-structured interviews with service users, carers and care coordinators; and care plan reviews at four NHS Trusts in England and two local Health Boards in Wales that are commissioned to deliver community mental health services.

#### Participants

For the service user interviews help will be sought from the MHRN/ NISCHR CRC Clinical Studies Officers and Research Nurses. Lists of service users under the care of the selected CMHT and subject to the CPA/CTP, will be checked with the responsible psychiatrist or team leader to prevent inappropriate mailings. In each setting the final list of service users for sampling will be grouped into care coordinator categories to enable us to gain different service user/carer/care coordinator triads. Any care coordinators already interviewed as senior practitioners will be excluded. From the remaining lists, up to four service users per care coordinator will be randomly selected and letters will be posted to them inviting them to contact the research team by phone, post or email if they wished to participate in an interview about their experiences of care planning and coordination. Once a service user contacts the team, a researcher will explain the study, answer any questions and arrange a date, time and venue for the interview. If insufficient responses have been received within four weeks of the mail-out, a second batch of invitations will be posted. This will be repeated until the target number was met or time ran out.

#### Inclusion criteria

##### Service users

1. 18 years of age or over.
2. A history of severe mental illness.
3. Receiving community mental health care for at least six months under the auspices of the Care Programme Approach (CPA) or Care and Treatment Planning (CTP)
4. Able to provide informed consent to participate.
5. Sufficiently proficient in English or Welsh to understand the questions being posed to them or willing to participate with the aid of an interpreter.

##### Carers (Family members or friends)

1. 18 years of age or over.
2. Providing emotional and/practical support to service users.
3. Agreement from the service users to participate in the study.

##### Care coordinators

1. The care coordinator for the service user interviewed in the embedded case study.

#### Materials

Two sets of study materials will be developed: 1) interview schedules and 2) a care plan review template. These materials will be developed by the study team in consultation with our advisory groups, the PAG and LEAG and drawing on the relevant literature.

1. Two interview schedules will be developed, one for service users and a carer version. All interviews will aim to explore participants’ views and experiences of care planning and coordination, recovery and personalisation, safety and risk, and the context within which these operated. Interview schedules will include lead questions with numerous prompts suggested for the interviewer. Schedules will be piloted with our service user researchers and amended if required.
2. A care plan review template will be developed. The template will focus on the inclusion of the views, co-production, recovery, personalisation, self-management and advanced directives. Care plans will also be used to collate demographic, diagnostic and service use data.

#### Service User Researchers (SURs)

An integral part of this study is the involvement of service users and carers in the design and implementation. This study will fully commit to this by employing service users to work alongside the research team to help with recruitment and to interview service users and carers. Training and support will be provided and structured reflection methods will be employed to help both the service users and academic researchers to learn about the joint-experiences and improve their ways of working.

#### Procedure

1. On the acceptance of the invitation to interview the service user will be provided with further information about the study. The service user will be asked for the name of anyone they consider to be an informal carer that we might contact for interview. At the same time the Clinical Studies Officers will identify their care coordinator. It will be made clear that there would be no disclosure of shared information between parties and the care coordinator will not be told the specific service user had taken part in the interview. Service users will also be asked for permission to review their care plans.
2. Interviews will be arranged at the convenience of the service user and carer. Face-to-semi-structured interviews, based upon the interview schedule will be conducted by Service User Researchers (SURs). An academic researcher will be in attendance to take informed consent and facilitate the interview process if needed.
3. CPA/CTP Care plan reviews will be undertaken by Clinical Studies Officers (CSO) using a template provided. CSOs will be given training on how to complete the template and given guidance if any queries round relevance of information arise.

#### Analysis

The analysis plan for the service user and carer interviews follows the same plan as highlighted above for the senior manager and practitioner interviews. An additional step will be included in the analysis.

1. Summarised data from the embedded micro-level case studies at each site will be subject to further comparison of the views expressed by linked service users, carers and care coordinators. This data will be compared against the review of the care plan. This will allow us to tease out agreements and disagreements in the perspectives of the participants within these triads.

#### Sample size calculation

The sample size calculations for the interviews was based on informed estimations of the number of care coordinators per CMHT (six). Assuming half agree to take part, this then give us a suggested number of service users to randomly select from care coordinator caseloads (approximately 25 per care coordinator with a predicted response rate of 10 per cent ) for research interviews and care plan reviews, giving us a total of seven service users per CMHT. We aim to recruit six service users per team and where possible their associated informal carer and care coordinator.

#### Synthesis and integration of datasets

The Framework method will be employed to bring together charted summaries of qualitative data alongside summary statistics of the quantitative measures for each case study site, noting points of comparison and contrast between what we will find in our analysis of each type of data.

Armed with our set of six within-case analyses we will conduct a cross-case analysis to draw out key findings from across all sites. We will consider the relationships between stated orientations to recovery and personalisation in national and local policy and senior staff interviews, and what we will find by studying the accounts of users, carers and care coordinators and by reviewing written care plans. In this way we will be able to investigate the data to identify ‘evidence’ at the intersections between macro-meso-micro levels and CPA/CTP care planning, recovery and personalisation; hence the ‘transformative’ nature of the study design [28].

#### Ethical issues

The study gained NHS Research Ethics approval from the NRES Committee Yorkshire & The Humber – Sheffield (Ref: 13/YH/0056A) on 13th February 2013. A major amendment was approved on 7th May 2013 to allow a reminder letter to be sent to service users for the questionnaire component and for the interview invitation letter to include information about interviewees receiving a £15 payment.

All participants will be required to give informed consent to participate in the study. Considerable attention will be given to ensuring the welfare of service user, carer and other participants and of the researchers. This includes providing opportunities to pause or withdraw from interviews; assurances of anonymity and confidentiality and responding to concerns for people’s welfare. Careful arrangements will be made for the location and conduct of interviews and all researchers will receive training, supervision and opportunities for debriefing.

## Discussion

This cross-national comparative study of care planning and coordination in community mental healthcare settings will provide a unique investigation of everyday practices and experiences from the perspectives of a range of stakeholders across multiple sites and two countries. The employment of a range of methods coupled with the meta-narrative literature review and a detailed plan for within case and across analysis will provide a detailed picture of the state of play and enable the identification of areas for attention and further research.

We will frame our data analysis by drawing on social scientific ideas and the findings of our Phase 1 evidence and policy review, an approach used by co-investigators in previous studies [49]. Our concern to explore commonplace practices in community mental health is congruent with interactionist interests in social processes and human action [50]. This perspective also recognises the importance of social structures, so that in any given setting person-to-person negotiations are shaped by features of organisational context [51]. The immediate context for frontline practitioners/care coordinators in this study is the CMHT workplace, each of which we view as a complex open system. Each participating team also sits within a larger meso-level NHS Trust/Health Board site, which in turn is located within a national-level system of mental health services. This idea of ‘nested systems’ is a feature of complexity thinking [37], and will inform our plan to generate, analyse and connect data at different (but interlocking) macro/meso/micro ‘levels’ of organisation. Analysis and interpretation of the case study data will be informed by a conceptual framework that emphasises the connections between different (macro/meso/ micro) levels of policy and service organisation, and that draws on the findings of the literature and national policy review in relation to care planning, recovery and personalisation.

The strengths of the study include data collection from a wide range of participants using a mix of methods from across a reasonable spread of teams and service providers in geographically varied locations in two countries. The framework method will provide a structured method of comparing data across research sites with local policies. This protocol was developed with a high level of service user and carer involvement, including an independent service user researcher as co-investigator. Further consultation was sought from an NIHR-funded service user and carer group advising research (SUGAR), LEAG and the PAG. Service user researchers will be employed to work alongside the research team. There is evidence that service user researchers both collect and interpret qualitative data differently from conventional researchers and in a way that is more in tune with the priorities of service users themselves [52, 53].

## Conclusion

With this study we aim to identify and describe the factors that ensure care planning and coordination in community mental health services is personalised, recovery-focused and conducted collaboratively. We will seek to address this aim by using a mixed methods approach examining data at a macro, meso and micro level, within and between cases. The findings from this study will enable us to make recommendations for service commissioners and providers and identify areas for future research.

## Abbreviations

COCAPP: Collaborative care planning project
CPA: Care programme approach
CTP: Care co-ordination and care and treatment planning
MNM: Meta-narrative mapping
NHS: National health service
ASSIA: Applied social sciences index and abstracts
CINAHL: Cumulative index to nursing & allied health literature
AMED: The Allied and complementary medicine database
EMBASE: Excerpta medica database
ERIC: Education resources information center
BHI: British humanities index
CMHT: Community mental health team
PAG: Project advisory group
LEAG: Lived experience advisory group
CQC: Care quality commission
SUR: Service user researcher
RSA: Recovery self-assessment scale
STAR-P: The scale to assess the therapeutic relationship
ES: The empowerment scale
MHRN: Mental health research network
NISCHR CRC: National institute for social care and health research clinical research centre
CSO: Clinical studies officer
NIHR: National institute for health research
